# Age-Related Decline in Intestinal Villus Length: A Cross-Sectional Study on the Human Gut

**DOI:** 10.3390/nu18081172

**Published:** 2026-04-08

**Authors:** Francisco Vara-Luiz, Carolina Palma, Ivo Mendes, Francisco Piçarra, Ana Elisa Teles, Filipe Nogueira, Inês Costa-Santos, Gonçalo Nunes, Marta Patita, Irina Mocanu, Sara Pires, Tânia Meira, Ana Vieira, Pedro Pinto-Marques, Paulo Mascarenhas, Iryna Leskiv, Daniel Gomes-Pinto, Jorge Fonseca

**Affiliations:** 1 Gastroenterology Department, Hospital Garcia de Orta, 2805-267 Almada, Portugal; 2 Aging Lab, Egas Moniz Center for Interdisciplinary Research (CiiEM), Egas Moniz School of Health and Science, 2829-511 Almada, Portugal; 3 Pathology Department, Hospital Garcia de Orta, 2805-267 Almada, Portugal

**Keywords:** aging, duodenal mucosa, histology, villus length, clinical nutrition

## Abstract

**Background/Objectives**: There is widespread agreement that age is a significant predictor of impaired response to nutritional support. This is generally attributed to anabolic resistance, with impaired absorption considered irrelevant/non-existent. However, animal models demonstrate age-related structural changes in the intestinal mucosa that may reduce absorptive capacity. We aimed to evaluate potential histological changes in the duodenal mucosa associated with aging. **Methods**: We conducted a single-center observational cross-sectional study. Ambulatory younger (18–45 years) and older (≥70 years) adults referred for upper endoscopy were included and underwent duodenal biopsies. Those biopsies were analyzed and compared for histological/histomorphometric changes, including villus length. Clinical and laboratory data were also recorded. **Results**: One hundred patients were included (46 men/54 women), 50 aged 18–45 years and 50 aged ≥70 years. There were no duodenal endoscopic changes. The median villus length was 0.35 mm (IQR 0.32–0.41 mm) in older people, lower than in younger adults (0.57 mm; IQR 0.47–0.68 mm) (*p* < 0.001). In a multivariable regression model including age, sex, and Charlson comorbidity index, age remained inversely associated with villus length (*p* < 0.001). Older participants also exhibited lower hemoglobin, iron, folate, vitamin B12, albumin and vitamin D levels, despite normal inflammatory markers. **Conclusions**: Aging is associated with histological changes in the intestinal mucosa, including villus shortening. These findings support the concept of mucosal aging as a distinct biological process. Villus shortening may reflect reduced absorptive surface area and could contribute to age-related nutritional vulnerability, although its functional implications remain to be determined.

## 1. Introduction

Normal gastrointestinal function is essential for nutrient digestion and absorption, drug bioavailability and protection against ingested pathogens, representing the largest interface between the host and the external environment. These processes depend on coordinated motility, secretion and mucosal integrity, under regulation by the enteric nervous system [1].

Aging is a complex biological process associated with structural and functional changes across multiple organ systems, including the gastrointestinal tract [2,3]. Age-related alterations have been described in pancreatic [4] and hepatic [5] function and colonic motility [6]. However, the structural and cellular hallmarks of small intestinal mucosal aging remain poorly defined. The small intestine is the primary site of nutrient absorption [7], drug uptake and host–microbiota interaction [8], yet it has received limited attention as an independent target of age-related remodeling. Moreover, older individuals are frequently underrepresented in research studies [9], contributing to the limited understanding of intestinal mucosal changes in this population.

Malnutrition is highly prevalent among older adults [10], affecting approximately 5% of community-dwelling senior citizens, 20–40% of nursing home residents, and up to 50% of hospitalized older patients [11]. While malnutrition in aging is traditionally attributed to reduced intake, comorbidities, and systemic metabolic alterations [12,13], the potential contribution of impaired intestinal absorptive capacity remains insufficiently explored.

Age is a strong predictor of reduced response to nutritional interventions [14], as older individuals exhibit diminished gains in body weight, lean mass, and protein synthesis despite adequate caloric and protein supplementation [15,16]. This phenomenon is largely explained by anabolic resistance related to chronic low-grade inflammation, insulin resistance, and increased splanchnic extraction of amino acids [17,18], whereas intestinal malabsorption is generally considered negligible. However, experimental studies challenge this assumption. In rodent models, aging is associated with the degeneration of villi, reductions in villus height and density, mucosal thinning and decreased enteric neuronal density, particularly within the myenteric plexus [19,20,21]. Age-related epithelial barrier dysfunction and alterations in epithelial turnover and mitochondrial function have also been described [22,23]. However, evidence of structural mucosal changes in humans is scarce and contradictory [24]. Whether the intestinal mucosa undergoes intrinsic age-related remodeling, independent of overt disease, remains unclear.

Establishing structural correlations of intestinal aging is critical to understanding the potential contribution of the gut to age-related syndromes such as malnutrition, frailty, and micronutrient deficiency. The present study aimed to evaluate potential age-related histological changes in the duodenal mucosa in older people.

## 2. Materials and Methods

### 2.1. Study Design

We conducted a single-center observational cross-sectional study at a tertiary hospital. The institutional ethics committee approved the study under authorization 71/2023, 8 September 2023. Because the study was non-interventional, clinicians managed patients according to routine practice.

### 2.2. Patients

Consecutive younger (18–45 years) and older (≥70 years) adults referred for upper endoscopy for indications unrelated with intestinal alterations were initially included. Age groups were defined a priori as 18–45 years and ≥70 years to enhance discrimination between younger adult and advanced aging mucosa, while minimizing biological overlap from middle-aged individuals, in whom age-related intestinal remodeling could be more variable. Additional inclusion criteria included providing informed consent to participate in the study.

Exclusion criteria included:

1. Pregnancy.
2. Clinical symptoms that may reflect malabsorption (nausea, vomiting, diarrhea and/or unintentional weight loss).
3. Clinical features suggestive of malnutrition, including anorexia and reduced/insufficient oral intake.
4. Known gastrointestinal diseases affecting the small bowel (e.g., celiac disease and inflammatory bowel disease).
5. Oncologic disease.
6. Drugs that may result in enteropathy (mycophenolate mofetil, colchicine, olmesartan, losartan, and non-steroid anti-inflammatory drugs).
7. Chemoradiation and/or immunomodulatory therapy.
8. Technical impossibility to obtain adequate intestinal biopsies.
9. Histologic diagnosis of pathogenic agents (including Helicobacter pylori) or other intestinal disorders not identified during upper endoscopy.

All participants signed an informed consent form. For each patient enrolled, the following methodology was applied:

1. Clinical and demographic database registry that includes age, gender, clinical indication for upper endoscopy, medication and laboratory data.
2. Upper endoscopy was performed by one gastroenterologist with the patients under deep sedation. The procedure was performed according to several quality indicators, including the use of a mucosa visibility score—Gastroscopy Rate of Cleanliness Evaluation (GRACE)—and preprocedural simethicone [25]. Endoscopic diagnoses were coded and collected in a predefined, standardized and searchable fashion. Antithrombotic therapy was managed according to international guidelines [26].
3. Collection of 2–3 duodenal biopsies (second/third portion) with standard biopsy forceps, which were then analyzed and compared for histological and histomorphometric changes.

### 2.3. Duodenal Biopsy Analysis

The duodenal biopsies were analyzed by two pathologists, including one expert gastrointestinal pathologist, and compared for histological changes. Interobserver agreement between pathologists was assessed using Cohen’s kappa coefficient.

Histological analysis was performed using hematoxylin–eosin (H&E) staining. Standard parameters assessed included villus architecture, crypt depth, epithelial integrity, and inflammatory infiltrates. All samples were analyzed for the presence of mucosal atrophy according to the adapted Marsh–Oberhuber classification [27]. For quantitative analysis, digital images of H&E-stained sections were acquired using a light microscope equipped with a high-resolution camera. Villus length was measured using Ventana DP 200 (Ventana Medical Systems^®^, Tuckson, AZ, USA). For each sample, at least three well-oriented villi were measured, and median values were calculated. Only well-oriented villi were included for morphometric analysis, defined as villi with a clearly visible longitudinal axis, intact epithelial lining and a visible connection to the crypt–villus junction.

### 2.4. Sample Size and Statistical Analysis

Because this was a single-center observational study involving duodenal biopsies obtained during clinically oriented upper endoscopy, a formal a priori sample size calculation was not performed. Instead, a pragmatic sample size was adopted, based on feasibility, on previous studies and on consecutive recruitment during the study period. Inclusion of 50 participants per group was considered sufficient to estimate and compare the primary morphometric parameter (villus length) between younger and older adults, while preserving balanced age-group representation.

The statistical analysis was performed using the Statistical Package for Social Sciences (version 28; IBM Corp., Armonk, NY, USA) and Microsoft Excel (version 16.89.1, 2024). Normality was assessed using the Kolmogorov–Smirnov test. Continuous variables were expressed as the mean ± standard deviation (SD) or median (interquartile range [IQR]), depending on data distribution. Categorical variables were expressed as frequencies and percentages. Between-group comparisons were performed using Student’s *t*-test or the Mann–Whitney test for continuous variables. Categorical variables were compared using the χ^2^ or Fisher’s exact test, as appropriate. Since laboratory parameters were not available for all patients, analyses were therefore performed using available data for each variable (complete-case analysis), and no imputation methods were applied. The number of observations was reported in the corresponding table.

Linear regression analysis was used to evaluate the association between age and duodenal villus length. A multivariable linear regression model was subsequently performed to assess whether this association remained independent after adjustment for sex and Charlson comorbidity index. Regression coefficients (β), 95% confidence intervals (CI), and *p*-values were reported. Model fit was evaluated using the coefficient of determination (R^2^) and adjusted R^2^. Model assumptions were assessed, including linearity, normality and distribution of residuals, and homoscedasticity. Collinearity between predictors was evaluated using variance inflation factors (VIFs). A *p*-value < 0.05 was considered statistically significant.

## 3. Results

### 3.1. Patients

**Figure 1:**
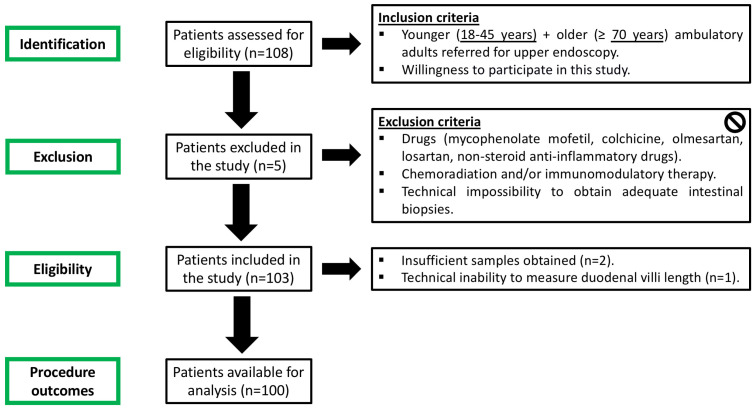
Patient flowchart in this study.

The patient flowchart of this study is detailed in Figure 1. A total of 108 patients were initially enrolled in the study and underwent upper endoscopy. Duodenal biopsies were taken during the procedure for histologic analysis. In total, 100 patients were available for complete analysis.

### 3.2. Baseline Patient Characteristics

A total of 100 patients who fulfilled the inclusion criteria were included in the study: 46 men/54 women, 50 aged 18–45 years and 50 aged ≥70 years. Baseline demographic and clinical characteristics were comparable between groups with respect to sex distribution and clinical indication for upper endoscopy, as described in Table 1. Older adults had a higher comorbidity burden, as reflected by the Charlson comorbidity index. Intake of proton pump inhibitors did not differ between younger (*n* = 21, 42%) and older adults (*n* = 23, 46%). No patient was under iron therapy or vitamin supplements. The most frequent indication was dyspepsia (*n* = 34), followed by gastroesophageal reflux disease (*n* = 32) and peptic ulcer disease (*n* = 19). No post-procedural adverse events were recorded. Older adults presented significantly lower hemoglobin (*p* = 0.029), iron (*p* = 0.038), folic acid (*p* = 0.027), vitamin B12 (*p* = 0.044), albumin (*p* < 0.001) and vitamin D (*p* = 0.034). No differences were found in between groups with respect to ferritin, transferrin saturation, total iron binding capacity (TIBC), C-reactive protein (CRP) and fecal calprotectin. All included patients had normal duodenal endoscopic evaluation, and no cause for anemia was detected in the upper gastrointestinal tract. None of the participants had endoscopic or histological evidence of celiac disease, inflammatory bowel disease, or other pathological conditions that could account for mucosal alterations.

### 3.3. Histological and Histomorphometric Findings

Duodenal biopsies of younger (18–45 years-old) and older (≥70 years-old) adults preserved the general villus–crypt architecture with no evidence of mucosal atrophy. No significant increase in lamina propria inflammatory infiltrate was observed, and epithelial integrity was preserved in all cases.

**Figure 2:**
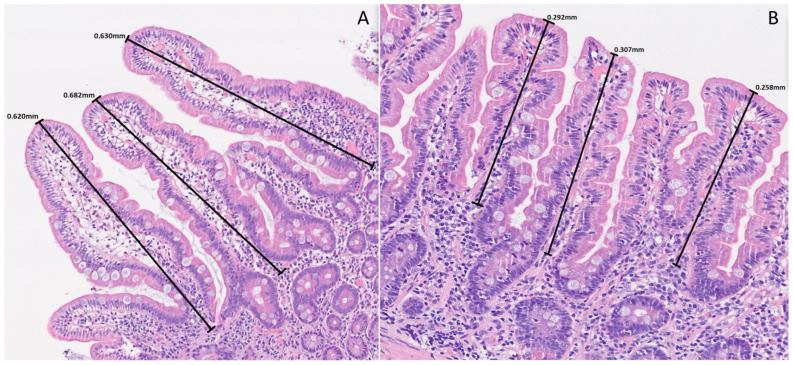
Duodenal mucosa histology stained with hematoxylin and eosin (magnification 150x) showing preserved villus–crypt architecture in younger (**A**) and older (**B**) adults. However, older individuals presented reduced villus length.

**Figure 3:**
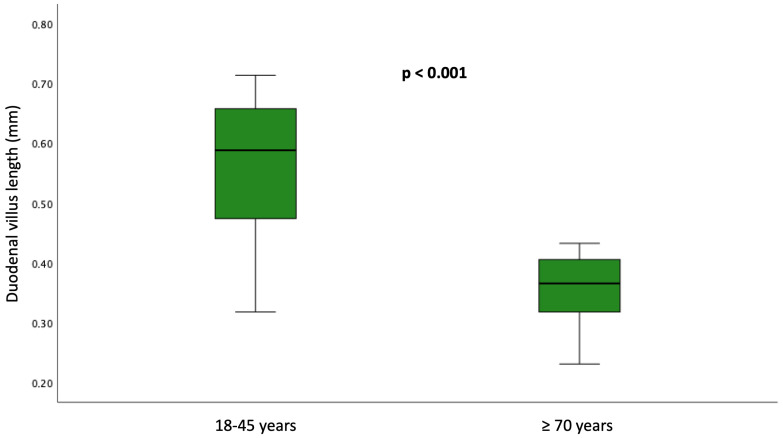
Boxplot comparing younger (*n* = 50) and older (*n* = 50) adults and demonstrating significantly shorter villus length in the older group (*p* < 0.001).

The median villus length was 0.35 mm (IQR 0.32–0.41 mm) in older people, lower than in younger adults (0.57 mm; IQR 0.47–0.68 mm) with a significant difference (*p* < 0.001). Nevertheless, in the younger group, 12 patients (24.0%) displayed villus length below 0.5 mm. Crypt depth did not differ significantly between groups. The global inter-observer agreement (Kappa) between pathologists was almost perfect (0.92). Figure 2 illustrates the histological and morphometric findings, and Figure 3 displays the boxplot showing significant differences.

**Figure 4:**
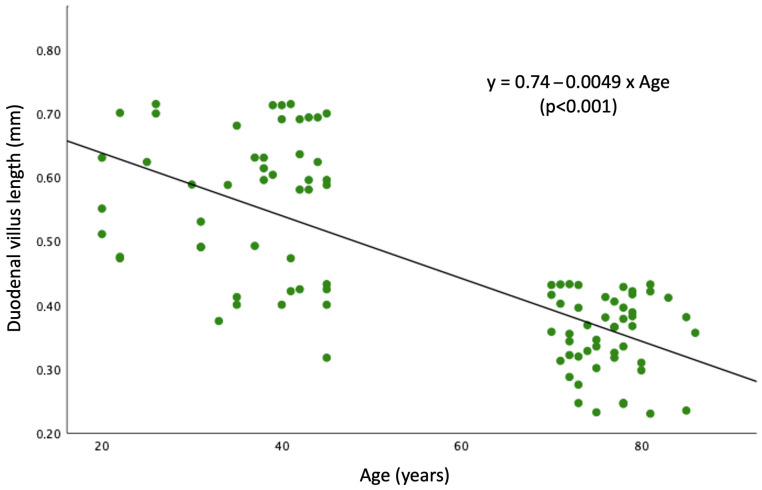
Linear regression model showing the inverse association between age and duodenal villus length within the studied bimodal age range. Each point represents a patient (*n* = 100). The black line shows the fitted regression line (slope = −0.0049; *p* < 0.001).

Linear regression analysis (Figure 4) demonstrated a significant inverse association between age and duodenal villus length (β = −0.0049 mm/year; 95% CI −0.006 to −0.004; R^2^ = 0.35; *p* < 0.001). The model indicates an average reduction of approximately 0.05 mm in villus length per decade within the studied bimodal age range. The regression equation was:

Duodenal villus length = 0.74 − 0.0049 × Age

In a multivariable linear regression model including age, sex, and Charlson comorbidity index, age remained independently associated with duodenal villus length (β = −0.008 mm/year; 95% CI −0.011 to −0.005; *p* < 0.001). Charlson comorbidity index was also associated with villus length (β = −0.069; 95% CI –0.095 to −0.042; *p* < 0.001), whereas sex was not significantly associated with villus length (*p* = 0.795). The overall regression model was statistically significant (F = 10.16; *p* < 0.001) and explained 49.9% of the variability in villus length (R^2^ = 0.499; adjusted R^2^ = 0.475).

## 4. Discussion

In this study, we demonstrate that aging is associated with measurable morphometric changes in the human duodenal mucosa in a clinically relevant outpatient population without overt intestinal disease. Older adults exhibited significantly shorter villus than younger individuals, despite preserved crypt depth, epithelial integrity, and absence of inflammatory infiltrates. Importantly, these findings were observed in patients without intestinal disease, suggesting that villus shortening may represent an intrinsic feature of mucosal aging rather than a consequence of inflammatory or structural pathology.

The growing proportion of older individuals worldwide has intensified interest in the physiological processes underlying aging [28]. Animal studies have consistently shown reduced epithelial proliferation and stem cell dysfunction [29], villus atrophy and histomorphometric alterations [30,31], and mitochondrial impairment in the aging intestine [32,33]. In contrast, human data remain limited and conflicting, with heterogeneous results regarding villus height, crypt depth, villus–crypt ratio, and enterocyte morphology [34,35,36,37].

In our cohort, classical criteria for mucosal atrophy according to the Marsh–Oberhuber classification were absent in both groups, underscoring that aging-related changes may occur below the threshold of conventional histopathological definitions. Because conventional criteria may miss subtle age-related remodeling, we explored villus length as a surrogate morphometric marker, as previously suggested for refeeding syndrome [38]. The normal villus length in humans is not clearly established, with other studies reporting expected variation between 0.4 mm and 1 mm [39,40]. This variability likely reflects methodological and anatomical factors, including biopsy orientation, sectioning plane, and differences in measurement techniques, as well as regional variation along the small intestine. In particular, suboptimal orientation can lead to underestimation of villus length, underscoring the importance of standardized morphometric assessment. Notably, 70% of adults aged ≥70 years presented a median villus length below 0.4 mm, and regression analysis demonstrated a significant inverse association between age and villus length. However, the regression analysis should be interpreted in the context of the study design. As participants were stratified into two distinct age groups, the age distribution was bimodal rather than continuous. Therefore, the observed association should not be interpreted as reflecting a smooth life-course decline in villus length, but rather as supporting a difference between younger and older adults within this cohort.

Older adults presented significantly lower serum levels of hemoglobin, iron, folic acid, vitamin B12, albumin, and vitamin D levels, despite normal inflammatory markers (CRP, ferritin, fecal calprotectin, and TIBC). The absence of systemic inflammation and preserved iron storage parameters argue against anemia of chronic disease and suggest subtle absorptive inefficiency, although it was not directly assessed in our study. In clinical practice, the concordance between morphometric and laboratory findings supports the concept of structural mucosal aging. These findings may be consistent with age-related nutritional vulnerability, including micronutrient deficiencies and protein undernutrition observed in older adults. The potential clinical implications of these alterations, particularly in the context of chronic disease or acute illness [41,42,43], remain to be determined. Although these findings may be associated with reduced absorptive capacity, they should be interpreted with caution as they are not specific for impaired duodenal absorption. Thus, these biochemical alterations should be considered supportive, but not definitive, evidence of impaired absorption, and alternative explanations including reduced dietary intake, medication exposure, comorbidity burden, chronic disease and general frailty cannot be excluded.

Recent evidence highlights the complex interplay between aging, nutritional status, and systemic vulnerability in hospitalized older patients. Even in the absence of overt gastrointestinal disease, decreased levels of albumin (<3 g/dL) and hemoglobin (<11 g/dL) should alert medical professionals to potential malnutrition in hospitalized patients [44]. In this context, structural alterations of the intestinal mucosa, such as villus shortening, may represent an additional and previously underrecognized contributor to impaired nutrient handling and increased susceptibility to deficiency. Future research in hospitalized older patients may help to clarify this knowledge gap.

Malnutrition in older adults is a major public health concern and is strongly associated with frailty, sarcopenia, functional decline, and increased mortality [45]. While anabolic resistance remains a central mechanism explaining reduced response to nutritional support [46], our findings suggest that intestinal structural remodeling may also contribute. From a structural perspective, villus shortening may reduce the absorptive surface area [47,48,49] which could potentially be associated with impaired nutrient assimilation, although absorption was not directly assessed.

From a translational and clinical perspective, recognizing and characterizing human intestinal mucosal aging may have important implications. Structural alterations of the duodenal epithelium may influence not only nutrient absorption but also drug bioavailability and epithelial barrier function, which should be further assessed in future research. These processes may be relevant in the context of systemic syndromes of aging, such as frailty, sarcopenia, micronutrient deficiencies, and altered drug absorption/pharmacokinetics [50]. These findings highlight the importance of further research to better understand the clinical implications of intestinal mucosal aging in older adults.

The strengths of this study include standardized biopsy acquisition, strict exclusion criteria to minimize confounding intestinal pathology, age-stratified analysis, and high interobserver agreement. Moreover, our observational study provides a comprehensive analysis of younger and older patients referred to upper endoscopy in a real-world clinical setting. The use of regression analysis further supports the observed association within the study sample. Furthermore, the use of clearly separated age groups likely increased the ability to detect morphometric differences by minimizing overlap between transitional and advanced aging phenotypes. However, some limitations should be acknowledged. The study population consisted of patients undergoing clinically indicated upper endoscopy and does not represent malnourished individuals, frail hospitalized patients, or those receiving structured nutritional therapy, introducing a potential selection bias. Although participants did not have overt intestinal disease and strict exclusion criteria were applied, they may also not be fully representative of a healthy community population. Therefore, the generalizability of these findings to the broader population should be interpreted with caution. However, endoscopic procedure and biopsy sampling without clinical indication would not be ethically justifiable. Second, histological assessment was not blinded to clinical data, which may introduce potential interpretative bias and affect morphometric assessment. Third, a formal assessment of nutritional status was not performed. Nevertheless, participants did not present clinical features suggestive of malnutrition, and individuals with reduced oral intake or unintentional weight loss were excluded. In addition, laboratory parameters related to nutritional status, including hemoglobin, albumin, iron, folate, vitamin B12, and vitamin D, were available and analyzed. Fourth, functional absorption tests were not performed; therefore, the relationship between villus shortening and absorptive capacity remains indirect. Finally, an important consideration in the interpretation of our findings is the potential role of confounding factors. Older participants presented a higher comorbidity burden, and all patients were recruited in a clinical setting based on indications for upper endoscopy. Therefore, comorbidities, indication bias, and age-related clinical factors, including medication exposure, may have influenced the observed differences in villus length. Although we applied strict exclusion criteria to minimize known causes of enteropathy and adjusted for comorbidity burden in multivariable analysis, residual confounding cannot be fully excluded. Nevertheless, the persistence of the association between age and villus length after adjustment supports the hypothesis that aging itself may be associated with structural remodeling of the intestinal mucosa. Future studies integrating standardized nutritional evaluation and ultrastructural, molecular, and functional assessments are warranted to clarify the mechanistic basis and its potential clinical impact.

## 5. Conclusions

In conclusion, this study suggests that aging is associated with histological changes in the intestinal mucosa, including villus shortening. These findings support the concept of mucosal aging as a distinct biological process. Villus shortening may reflect reduced absorptive surface area and could contribute to age-related nutritional vulnerability, although its functional implications remain to be determined.

## Figures and Tables

**Table 1:** Baseline patient characteristics. Data are presented as the mean ± standard deviation or *n* (%) as appropriate. The number of observations for each laboratory variable is indicated in parentheses. SD—standard deviation.

Variables		18–45 Years (*n* = 50)	≥70 Years (*n* = 50)	*p* Value
Age		34.8 ± 8.2 years	76.2 ± 4.1 years	
Gender	Male	24 (48.0%)	22 (44.0%)	0.401
	Female	26 (52.0%)	28 (56.0%)	
Charlson comorbidity index		0.2 ± 0.4	3.8 ± 1.6	0.014
Clinical indication for upper endoscopy	Dyspepsia	17 (34.0%)	17 (34.0%)	0.389
	Gastroesophageal reflux disease	12 (24.0%)	10 (20.0%)	0.337
	Peptic ulcer disease	10 (20%)	9 (18.0%)	0.480
	Dysphagia	4 (8.0%)	3 (6.0%)	0.363
	Gastric antral vascular ectasia	0 (0.0%)	2 (4.0%)	0.106
	Radiological findings	3 (6.0%)	4 (8.0%)	0.492
	Gastric polyp	2 (4.0%)	3 (6.0%)	0.062
	Iron deficiency anemia	2 (4.0%)	2 (4.0%)	0.390
Laboratory evaluation	Hemoglobin (g/dL)	13.6 ± 1.3 (50)	12.9 ± 1.7 (50)	0.029
	Iron (µg/dL)	88.5 ± 28.3 (50)	73.1 ± 32.6 (50)	0.038
	Ferritin (ng/mL)	134 ± 94 (44)	162 ± 120 (43)	0.086
	Transferrin saturation (%)	22.9 ± 9.1 (39)	25.6 ± 9.5 (39)	0.161
	Total iron binding capacity (µg/dL)	62.9 ± 12.3 (39)	59.1 ± 13.3 (39)	0.149
	Folic acid (ng/mL)	6.4 ± 2.9 (39)	5.1 ± 3.8 (41)	0.027
	Vitamin B12 (pg/mL)	650 ± 53.8 (39)	549 ± 69.5 (41)	0.044
	Albumin (g/dL)	4.5 ± 0.5 (43)	3.5 ± 0.6 (41)	<0.001
	C-reactive protein (mg/dL)	1.45 ± 2 (50)	1.2 ± 1.2 (50)	0.144
	Vitamin D (ng/mL)	23.8 ± 6.4 (29)	16.3 ± 10.9 (32)	0.034
	Fecal calprotectin (µg/g)	22.7 ± 21.9 (32)	39.3 ± 21.9 (30)	0.076

## Data Availability

All the data analyzed during this review are included in this article. Further inquiries can be directed to the corresponding author.

## Abbreviations

The following abbreviations are used in this manuscript:

CI	Confidence intervals
CRP	C-reactive protein
H&E	Hematoxylin–eosin
TIBC	Total iron binding capacity
